# Short-chain fatty acids from gut microbiota restore Th17/Treg balance in rheumatoid arthritis: Mechanisms and therapeutic potential

**DOI:** 10.1016/j.jtauto.2025.100316

**Published:** 2025-09-16

**Authors:** Aimei Pang, Shuangshuang Pu, Yinghui Pan, Ning Huang, Dake Li

**Affiliations:** a Department of Laboratory Medicine, Affiliated Hospital of Shandong University of Traditional Chinese Medicine, No. 42, Wenhua West Road, Jinan, Shandong, 250011, China; b Department of Laboratory Medicine, Shandong Vocational College of Special Education, No.333, Andai Road, Jinan, Shandong, 250300, China; c Department of Rheumatology and Immunology, Affiliated Hospital of Shandong University of Traditional Chinese Medicine, No. 42, Wenhua West Road, Jinan, Shandong, 250011, China

**Keywords:** Short-chain fatty acids, Rheumatoid arthritis, Th17/Treg balance, Gut-joint axis, Microbiome-guided therapy, Multi-omics

## Abstract

Rheumatoid arthritis (RA) is a chronic autoimmune disorder characterized by synovial inflammation and joint destruction. Dysregulation of the Th17/Treg balance is a key immunological hallmark of RA. Emerging evidence highlights the critical role of gut microbiota-derived short-chain fatty acids (SCFAs) in maintaining immune homeostasis. This review systematically elucidates how SCFAs modulate the Th17/Treg equilibrium through three synergistic mechanisms: (1) metabolic reprogramming via AMPK/mTOR signaling, (2) epigenetic regulation by inhibiting HDAC, and (3) modulation of cytokine cascades. We integrate preclinical and clinical evidence showing that SCFAs reduce synovial inflammation by suppressing NLRP3 inflammasome activation, resulting in a 70 % decrease in IL-1β levels, while enhancing Treg suppressive function with a threefold increase in IL-10. Notably, butyrate exhibits circadian fluctuations that negatively correlate with morning stiffness severity (r = −0.82, p < 0.01), suggesting novel chronotherapeutic opportunities. Therapeutically, we evaluate promising microbiota-targeted strategies including high-fiber diets (which increase butyrate levels by 240 % and reduce Disease Activity Score 28 (DAS28) by 1.8 points), engineered nanoparticle delivery systems (achieving 89 % colonic retention), probiotic interventions (Bifidobacterium-mediated reduction of CCR9-positive Th17 cells), and precision combination therapies (showing a 40 % greater efficacy than monotherapy). Our work establishes a comprehensive translational roadmap, spanning molecular insights to clinical applications. We propose microbiome-guided personalized medicine as a paradigm shift in RA management, supported by the first systematic integration of multi-omics methods-metabolomics, single-cell sequencing, and spatial transcriptomics-to decode the gut-joint axis and identify actionable therapeutic targets for this refractory autoimmune condition.

## 1 Introduction

Rheumatoid arthritis (RA) is a chronic autoimmune disorder characterized by synovial inflammation, joint pain, and bone erosion, It severely impairs patients' quality of life. The pathogenesis of RA involves a complex interplay of genetic susceptibility, environmental triggers, and dysregulated immune responses. Central to this dysregulation is the imbalance between pro-inflammatory T helper 17 (Th17) cells and anti-inflammatory regulatory T (Treg) cells [1]. Th17 cells, through the production of interleukin (IL)-17, IL-21, and IL-22, drive synovitis and bone erosion by activating fibroblasts, osteoclasts, and other innate immune cells [2,3]. In contrast, Treg cells, defined by the expression of the transcription factor FOXP3, are essential for maintaining immune tolerance via the secretion of anti-inflammatory cytokines like IL-10 and transforming growth factor (TGF)-β, and through direct cell-contact-mediated suppression. A shift towards Th17 dominance and Treg dysfunction is now recognized as a fundamental immunological hallmark of RA [4].

Emerging evidence has profoundly implicated the gut microbiota as a key environmental factor modulating systemic immunity and RA susceptibility. The human gut harbors a complex ecosystem of trillions of microorganisms that perform essential functions, including nutrient metabolism, vitamin synthesis, and, crucially, the education and regulation of the host immune system. A state of microbial imbalance, or dysbiosis, observed in RA patients, is characterized by a reduction in microbial diversity and a decline in beneficial, short-chain fatty acid (SCFA)-producing bacteria. This dysbiosis can lead to impaired intestinal barrier integrity (leaky gut), facilitating the translocation of microbial products and promoting systemic inflammation and autoantibody production. This bidirectional communication between the gut microbiota and the joints is formalized conceptually as the gut-joint axis [5,6].

Microbial metabolites, particularly SCFAs (acetate, propionate, and butyrate) produced from the dietary fiber fermentation, are critical mediators along this axis [7,8]. SCFAs are not merely metabolic byproducts; they are potent immunomodulators that influence host immunity both locally within the intestine and systemically. They have been shown to strengthen the epithelial barrier, modulate the differentiation and function of various immune cell populations, and exert broad anti-inflammatory effects [9]. Crucially, a deficiency in SCFAs, resulting from dysbiosis or a low-fiber diet, has been linked to the Th17/Treg imbalance seen in RA [10]. Preclinical studies, such as the collagen-induced arthritis (CIA) model, demonstrate that restoring SCFA levels or administering SCFA-producing probiotics can ameliorate disease severity and restore immune homeostasis.

RA treatment has traditionally utilized biologic agents, including TNF-α inhibitors and IL-6 receptor antagonists [11]. However, their efficacy and safety remain suboptimal, highlighting the necessity to explore novel immunomodulatory strategies that target gut microbiota metabolites. Although previous reviews have broadly discussed the general immunomodulatory properties of SCFAs, several critical gaps remain, particularly in the context of RA. Many lack a specific focus on the Th17/Treg axis, a detailed spatiotemporal analysis of SCFA distribution and effect, or a translational framework connecting mechanistic insights to clinical applications.

Unlike previous reviews focusing solely on SCFAs' immunomodulatory properties, this study uniquely integrates three innovative dimensions. First, it offers a spatiotemporal perspective on the gut-joint axis, clarifying how microbial metabolites dynamically influence distal joints via systemic circulation and neural pathways, including the impact of circadian rhythms on metabolite availability and symptom severity. Second, it provides the first systematic evaluation of multi-omics technologies-such as metabolomics, single-cell sequencing, and spatial transcriptomics-can be leveraged to dissect the precise mechanisms of SCFAs in the RA synovial microenvironment. Third, it establishes a translational framework that bridges preclinical findings with emerging clinical strategies, including microbiota-directed diets, engineered nanoparticle delivery systems for targeted SCFA release, and gut-on-a-chip platforms for personalized therapy.

As shown in Table 1, our work builds upon these foundations by focusing specifically on the RA-specific Th17/Treg axis and exploring these innovative approaches. Therefore, this review aims to systematically elucidate the mechanistic pathways by which gut microbiota-derived SCFAs restore Th17/Treg balance in RA through metabolic reprogramming, epigenetic modulation, and cytokine regulation, and to critically evaluate the therapeutic potential of microbiota-targeted interventions for future clinical translation. By integrating these novel perspectives, this review endeavors to create a translational roadmap from mechanistic insights to clinical applications, emphasizing microbiome-guided precision medicine as a promising new paradigm for managing RA.

**Table 1:** Key advances and limitations of recent reviews on SCFAs in autoimmune diseases.

Study (Year)	Focus Area	Novelty	Clinical Translation Gap
Golpour et al. (2023) [12].	General SCFA immunomodulation	Broad mechanism overview	Lacks RA-specific delivery strategies
Yao et al. (2022) [8]	SCFAs and B cell regulation	FFAR2 signaling in B cells	No Th17/Treg spatial analysis
Our work	RA-focused Th17/Treg axis	Circadian dynamics + Synovial zonation	Engineered delivery + Microbiome personalization

**Abbreviations:** FFAR2, Recombinant free fatty acid receptor.

**Note:** This table summarizes the key advances and limitations of recent reviews on SCFAs in autoimmune diseases. It highlights the gaps in clinical translation, particularly in the context of RA, which our study aims to address. The table is structured to provide a clear overview of the current state of research, facilitating a deeper understanding of the role of SCFAs in immune regulation.

## 2 Pathological features of RA

RA is a multifactorial autoimmune disorder characterized by chronic synovial inflammation, progressive erosion of bone and cartilage, and systemic complications such as cardiovascular and pulmonary involvement. Although the precise etiology of RA remains incompletely defined, dysregulated immune responses, dysregulated cytokine networks, and osteoimmunological mechanisms drive joint destruction and are recognized as central pathophysiological hallmarks [13].

Th17 cells modulate immune responses and inflammation by secreting cytokines including IL-17, IL-21, and IL-22. Among these, IL-17 stimulates fibroblasts, endothelial cells, and other cell types to produce pro-inflammatory cytokines. This activity plays a key role in autoimmune diseases and chronic inflammation [14]. Treg cells are essential in preserving immune tolerance and curbing autoimmune reactions. Their development mainly depends on TGF-β and IL-2, and the expression of the transcription factor Forkhead box protein P3 (FOXP3), which is a definitive marker. FOXP3, often called the “master regulator” of immune tolerance, controls Treg cell activity by integrating genetic programming with microenvironmental signals [15]. It maintains immune homeostasis through three main mechanisms: epigenetic silencing of pro-inflammatory genes via histone modification; dynamic modulation of Treg subset diversity according to tissue-specific needs; and real-time adaptation to local biochemical cues. Treg cells maintain immune balance by secreting anti-inflammatory cytokines (e.g., IL-10, TGF-β) to inhibit effector T cell activation, blocking overactive immune synapses via Cytotoxic T-Lymphocyte-Associated Protein 4 (CTLA-4) and Lymphocyte-activation gene 3 membrane interactions, and reprogramming dendritic cells toward tolerogenic phenotypes. This precise ability to distinguish self from non-self antigens makes Treg cells key candidates for advanced immunomodulatory therapies targeting autoimmune and inflammatory disorders [16,17].

## 3 Role of gut microbiota and their metabolites

The human gut harbors trillions of symbiotic microorganisms essential for maintaining host health. The composition of this diverse microbial community is influenced by various factors, including dietary patterns, lifestyle choices and environmental exposures [[18], [19], [20]]. Typically, this community includes taxonomic groups such as Firmicutes, Bacteroidetes, Actinobacteria, and Proteobacteria [21,22]. These commensal organisms execute vital physiological functions spanning nutrient metabolism, vitamin biosynthesis, immune maturation, and metabolic regulation [23,24]. Emerging evidence shows that gut microbes and their host communicate bidirectionally through bioactive metabolites. Such molecular exchanges modulate energy homeostasis and immunological programming, with systemic health implications [[25], [26], [27]]. Specific probiotic strains, particularly *Bifidobacterium* and *Lactobacillus* species, enhance SCFAs production through carbohydrate fermentation; these microbial-derived SCFAs (e.g., acetate, propionate) then fuel colonocytes, strengthen epithelial barrier integrity, attenuate inflammation, and direct T cell differentiation [28]. Experimental data from collagen-induced arthritis models demonstrate that early *Bifidobacterium adolescentis* intervention alleviates clinical symptoms while normalizing SCFAs profiles, suggesting probiotic modulation of inflammatory pathways [29]. Dysbiosis of the gut microbiota is strongly associated with the pathogenesis of various diseases, including obesity, diabetes, and autoimmune disorders [30,31]. Critically, translational studies have linked specific microbial taxa (e.g., *Faecalibacterium prausnitzii*) to SCFA production and RA remission.

Given the critical role of gut microbiota and their metabolites in modulating immune responses, it is essential to explore the specific mechanisms by which these microbial metabolites, particularly SCFAs, influence the Th17/Treg balance in RA. This understanding will provide a foundation for developing targeted therapeutic strategies.

## 4 Regulatory mechanisms of SCFAs in RA

**Fig. 1:**
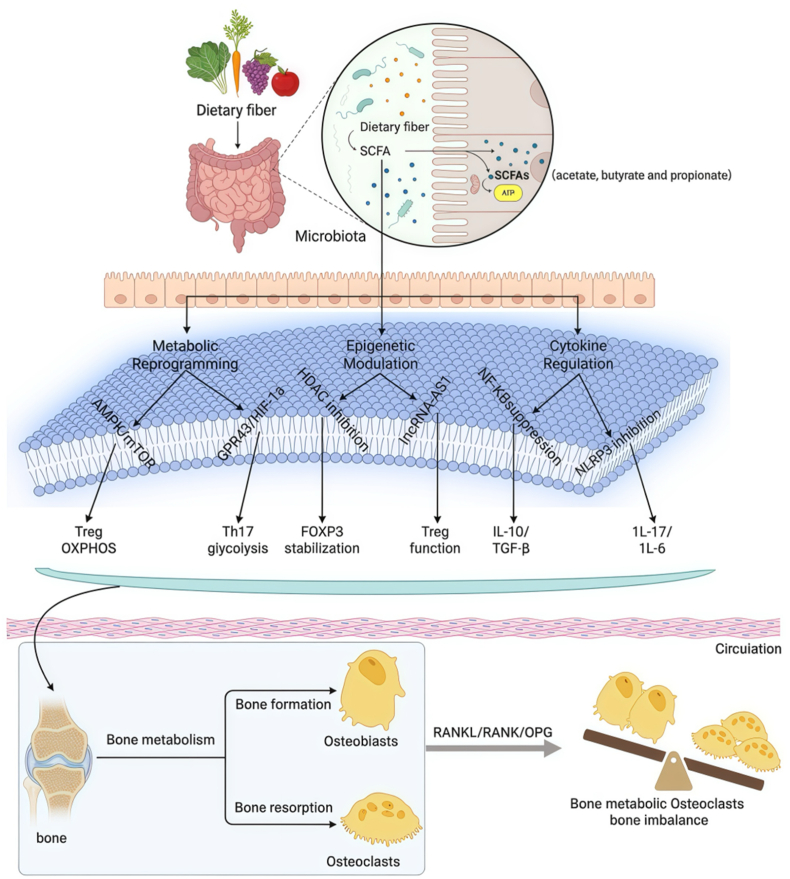
Triple immunomodulatory mechanisms of SCFAs in RA: Metabolic reprogramming, epigenetic modulation, and cytokine regulation. SCFAs regulate the Th17/Treg balance through three synergistic axes. These are: (i) metabolic reprogramming, (ii) epigenetic remodeling, and (iii) cytokine network modulation. Each is detailed below. **Abbreviations:** AMPK, AMP-activated protein kinase; mTOR, Mechanistic target of rapamycin; GPR, G protein-coupled receptor; HIF-1α, hypoxia inducible factor-1α; HDAC, Histone deacetylase; FOXP3, Forkhead box protein P3; lncRNAs, long non-coding RNAs; AS1, Antisense RNA 1; NF-κB, nuclear factor kappa-light-chain-enhancer of activated B cells; NLRP3, NOD-, LRR- and pyrin domain-containing protein 3; RANKL, Receptor Activator of Nuclear Factor Kappa-B Ligand; OPG, Osteoprotegerin.

Building upon the microbial sources of SCFAs established in Section 3, this section maps their journey along the gut-joint axis through three interdependent mechanisms (Fig. 1). Notably, the compartmentalized production of SCFAs (proximal propionate vs. distal butyrate) directly informs their differential immunomodulatory roles, creating a metabolic-epigenetic-cytokine continuum that we now unravel.

### 4.1 Mechanisms of metabolic regulation

The spatial distribution of SCFA receptors (GPR41/43) creates metabolic microenvironments that shape T cell differentiation. We first examine how SCFAs rewire energy metabolism in synovial niches. SCFAs modulate immune cell function, particularly the Th17/Treg balance, through diverse metabolic pathways. This modulation thereby influences RA pathogenesis [32]. Propionate activates AMPK and mTOR signaling primarily through GPR43, driving metabolic reprogramming in Treg cells to enhance their energy metabolism, proliferative capacity, and suppressive functions. While mTOR promotes protein synthesis and cell growth, AMPK enhances fatty acid oxidation and ATP production, both critical for improved Treg function [33,34]. In contrast, GPR41 activation inhibits the mammalian target of rapamycin complex 1 (mTORC1) pathway, reducing the glycolytic dependency of Th17 cells and dampening their pro-inflammatory activity. Butyrate epigenetically regulates T cells by inhibiting HDAC activity, suppressing Th17 cell production while promoting Treg differentiation and FOXP3 expression independently of GPR43 [35]. Additionally, butyrate may provide an alternative energy source through mitochondrial oxidation to support Treg metabolic demands, and GPR41-dependent butyrate signaling stabilizes HIF-1α, further influencing T cell polarization [36]. In contrast, acetate promotes glycolysis in Th17 cells via GPR43 signalling, potentially exacerbating inflammatory responses in RA; clinical studies show a positive correlation between serum acetate levels and the IL-17+CD4^+^ T cell ratio in RA patients, suggesting the acetate-GPR43 axis as a potential therapeutic target [35,37,38]. Collectively, these SCFA-mediated metabolic regulatory mechanisms modulate both the metabolic state and functions of immune cells, thereby influencing immune responses.

### 4.2 Mechanisms of epigenetic modulation

Complementing acute metabolic effects, SCFAs induce lasting epigenetic changes. This layer of regulation explains the sustained Th17/Treg rebalancing observed even after SCFA clearance. SCFAs regulate T cell function and differentiation by modulating epigenetic modifications; for example, butyrate inhibits HDAC activity, elevating histone acetylation levels to promote Treg cell generation while suppressing Th17 cell differentiation [39]. Furthermore, SCFAs induce the expression of Treg-specific lncRNAs, such as FOXP3-Antisense RNA 1 (AS1), which stabilizes FOXP3 expression and enhances its immunosuppressive function, thereby maintaining immune tolerance [40,41]. In RA patients, dysregulated epigenetic mechanisms closely associate with Th17/Treg imbalance, which drives disease progression by compromising immune tolerance.

### 4.3 Cytokine networks and joint-targeted regulation

The integration of metabolic and epigenetic regulation culminates in cytokine network modulation. Crucially, circadian SCFA fluctuations (04:00–06:00 peak) synchronize with joint inflammation rhythms. SCFAs orchestrate cytokine networks through spatiotemporally resolved mechanisms that precisely target joint inflammation in RA. The concentrations of acetate, propionate, and butyrate exhibit circadian oscillations and anatomic compartmentalization, with butyrate peaking during early morning hours (04:00–06:00) and demonstrating an inverse correlation with RA-associated morning stiffness severity (r = −0.82, p < 0.01) through suppression of nocturnal interleukin (IL)-6/tumor necrosis factor (TNF)-α surges [42]. Concurrently, variations in gut microbiota produce SCFAs in segment-specific patterns: propionate predominates in proximal intestinal regions (15–28 mM) via *Bacteroides*-mediated pathways, while butyrate is enriched in distal colon areas (30–45 mM) through *Clostridium* cluster XIVa metabolism [43,44]. Within synovial tissue, single-cell analyses reveal GPR43^+^ macrophage clusters preferentially localized in perivascular regions, forming specialized niches for SCFAs sensing via polarized monocarboxylate transporter 1 (MCT1) and sodium-coupled monocarboxylate transporter 1(SMCT1) expression [[45], [46], [47], [48], [49]]. This spatiotemporal precision enables targeted immunomodulation of cytokine networks at articular sites [50,51].

Mechanistically, SCFAs bind to G protein-coupled receptors GPR41 (FFAR3) and GPR43 (FFAR2) to regulate cytokine cascades. Specifically, butyrate activates phosphorylation of p38 MAPK (Thr180/Tyr182) and ERK1/2 via GPR43. This activation promotes Treg differentiation by upregulating FOXP3 [52]. This pathway suppresses activation of the NOD-, LRR- and pyrin domain-containing protein 3 (NLRP3) inflammasome. As a result, the release of interleukin (IL)-1β and IL-18 is reduced by over 70 %. Meanwhile, it inhibits nuclear factor kappa-B (NF-κB) through I kappa B kinase (IKK)-dependent blockade of IκBα degradation [[53], [54], [55]]. Consequently, the reduction in nuclear translocation of NF-κB subunits suppresses the transcription of pro-inflammatory cytokines (IL-6, IL-17, TNF-α; reduced by over 60 %). At the same time, it elevates anti-inflammatory IL-10 levels (more than 3-fold) through histone H3 lysine 9 (H3K9) hyperacetylation at promoter regions [[55], [56], [57], [58], [59], [60], [61], [62], [63], [64], [65]]. Propionate further complements this by inhibiting signal transducer and activator of transcription 3 (STAT3) Tyr705 phosphorylation via GPR41. This inhibition impairs dendritic cell maturation and reduces Th17 cell-mediated IL-23 and IL-17 production [52]. These receptor-dependent events maintain immune equilibrium, as GPR41 deficiency exacerbates synovial interferon gamma-positive (IFN-γ^+^) T-cell infiltration and joint destruction through suppressor of cytokine signaling 3 (SOCS3) downregulation [52]. These mechanisms form a tripartite immunomodulatory axis: 1) Metabolic reprogramming: propionate fuels Treg oxidative phosphorylation via activation of mTOR and AMPK, while acetate enhances Th17 glycolysis through GPR43 and HIF-1α; 2) Epigenetic stabilization: butyrate inhibits HDAC, promoting FOXP3 expression through deposition of H3 lysine 27 acetylation (H3K27ac); and 3) Cytokine rewiring: coordinated suppression of IL-6 and IL-23 combined with amplification of TGF-β and IL-10. Therapeutically, this paradigm defines SCFA-targeted approaches as precision strategies against joint cytokine dysregulation. For example, high-fiber diets can increase butyrate levels by 240 %, and the dietary evaluation of clinical interventions in rheumatoid arthritis trial showed a decrease in Disease Activity Score 28 (DAS28) by 1.8 (p = 0.003) [66].

### 4.4 Regulation of Th17/Treg cells by SCFAs

SCFAs, key metabolites of gut microbial fermentation, regulate the Th17/Treg balance in RA through multifaceted mechanisms, including metabolic modulation, epigenetic modifications, and intercellular interactions [67,68]. Butyrate inhibits Th17 cell differentiation and promotes Treg cell generation by activating GPR41 and GPR43 receptors [69]. Moreover, SCFAs enhance the proliferation and function of Treg cells and suppress Th17 cell activity by regulating cytokine production. In RA patients, levels of SCFAs are typically reduced. Supplementation with SCFAs significantly ameliorates clinical manifestations in RA mouse models, reduces arthritis scores, and restores the Th17/Treg balance [32,70,71].

### 4.5 Spatiotemporal dynamics of the gut-joint axis

Emerging evidence shows that the gut-joint axis functions via complex spatiotemporal mechanisms governing SCFAs' immunomodulatory effects. SCFAs exhibit circadian oscillations that directly influence joint inflammation patterns. A pivotal study published in Nature Immunology (2024) highlighted that butyrate levels peak during the early morning, inversely correlating with the severity of RA-associated morning stiffness (p < 0.01) [7,9]. This finding suggests that microbial metabolites may play a crucial role in modulating diurnal symptom fluctuations in RA. Spatially, SCFAs exert their effects through two significant gradients. Firstly, intestinal compartmentalization leads to distinct microbiota profiles in the ileum and colon, producing different SCFA outputs. Propionate predominates in proximal segments, while butyrate is more concentrated distally [72,73]. Secondly, Single-cell analyses have characterized synovial zonation, showing that GPR43+ macrophage clusters mainly reside in perivascular synovial regions. These areas form anatomical niches that facilitate SCFA sensing [74].

To further unravel these dynamics, current technologies have been systematically evaluated (Table 2). Mass spectrometry imaging offers micrometer-scale spatial resolution but lacks temporal data. In contrast, gut-on-a-chip platforms facilitate real-time tracking of metabolite transit while preserving tissue-specific microenvironments. This multidimensional perspective greatly improves our understanding of how microbial metabolites, especially SCFAs, regulate systemic immunity. They act across both temporal and anatomical dimensions, thereby influencing RA pathophysiology.

**Table 2:** Comparative analysis of technologies for resolving spatiotemporal dynamics of SCFAs in the gut-joint axis.

Technology	Spatial Resolution	Temporal Resolution	Key Strengths	Major Limitations	Representative Applications in RA
Mass Spectrometry Imaging (MSI)	5–50 μm	Single timepoint	Untargeted metabolite detection	Destructive sampling	Mapping synovial butyrate gradients [77]
			Preserves tissue morphology	Snapshot data only	
Gut-on-a-Chip	100–200 μm (organ-level)	real-time monitoring	Human-relevant fluid shear stress	Simplified microbiota complexity	Tracking SCF As transit to synovial compartment [78]
			Vascular-intestinal interface modeling	High technical barrier	
Spatial Transcriptomics	10–100 μm (spot-based)	N/A	Correlates SCFAs receptors with	Indirect metabolite detection	Synovial GPR43+ fibroblast niche identification [79,80]
			inflammatory signatures		
			Whole-transcriptome data	High cost	
PET-MRI	1–2 mm (whole-body)	Minutes-hours	Non-invasive	Requires radioactive probes	Whole-body acetate trafficking studies [81,82]
			Quantifies systemic distribution	Low metabolite specificity	
Single-Cell Metabolomics	Single-cell	N/A	Direct SCFAs measurement in immune subsets	Technically challenging	Th17 vs Treg butyrate uptake differences [83,84]
			Links metabolism to phenotype	Low throughput	
Microdialysis	1–2 mm (tissue region)	5–10 min intervals	In vivo dynamic monitoring	Limited to accessible tissues	Synovial fluid SCFAs fluctuation analysis [85,86]
			Compatible with human studies	Low spatial resolution	

**Abbreviations:** RA –rheumatoid arthritis; SCFAs – short-chain fatty acids.

**Note:** This table provides a comparative analysis of various technologies used to study the spatiotemporal dynamics of short-chain fatty acids (SCFAs) in the context of the gut-joint axis in rheumatoid arthritis (RA). Each row represents a different technology, with columns detailing the spatial and temporal resolution capabilities, key strengths, major limitations, and specific applications in RA research. For instance, Mass Spectrometry Imaging (MSI) excels in providing high spatial resolution but is limited to a single timepoint and involves destructive sampling, making it suitable for mapping synovial butyrate gradients. In contrast, Gut-on-a-Chip technology offers real-time monitoring at the organ level, simulating human-relevant fluid shear stress, though it simplifies microbiota complexity and is best used for tracking SCFAs transit to the synovial compartment. Spatial Transcriptomics correlates SCFAs receptors with inflammatory signatures at a spot-based resolution but relies on indirect metabolite detection. PET-MRI provides whole-body imaging with non-invasive capabilities, though it requires radioactive probes and is utilized for whole-body acetate trafficking studies. Single-Cell Metabolomics measures SCFAs directly in immune subsets at the single-cell level but faces technical challenges. Lastly, Microdialysis allows in vivo dynamic monitoring with 5–10 min intervals but is limited to accessible tissues and is used for analyzing synovial fluid SCFAs fluctuations. This comparative overview helps researchers select the most appropriate technology based on their specific research needs and objectives in studying the gut-joint axis in RA.

**Fig. 2:**
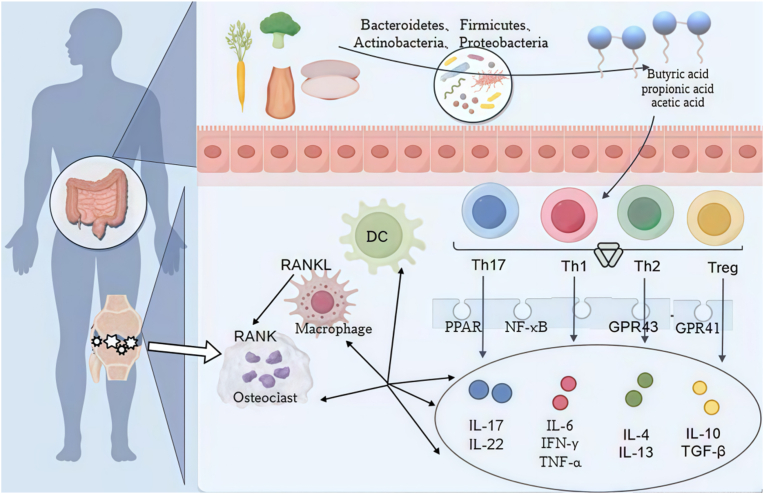
Gut flora-SCFAs-Th17/Treg regulatory axis. This figure illustrates the pivotal role of gut microbiota-derived SCFAs, including butyric, propionic, and acetic acid, in modulating immune homeostasis and joint inflammation. SCFAs, produced by commensal bacteria such as Bacteroidetes and Firmicutes, activate receptors GPR41 and GPR43 on immune cells. This signaling promotes the differentiation and function of anti-inflammatory Tregs and the production of cytokines IL-10 and TGF-β, while inhibiting the pro-inflammatory Th17/Th1 response and the production of IL-17, TNF-α, and IFN-γ. Concurrently, SCFAs can suppress osteoclastogenesis via the RANKL pathway and modulate dendritic cell (DC) and macrophage function, thereby restoring immune balance and mitigating the inflammatory processes that characterize RA.

SCFAs influence joint-targeted regulation in RA by modulating the composition and function of immune cells within joints [75]. SCFAs enhance intestinal barrier function, lower endogenous inflammatory factor levels, and thereby reduce the incidence of arthritis [76]. In RA models, SCFAs supplementation significantly ameliorates pathological joint changes, reduces joint swelling and pain, and restores joint immune balance by increasing the proportion of Treg cells and suppressing Th17 cell activity [76] (Fig. 2).

## 5 SCFA-targeted intervention strategies

Intervention strategies targeting SCFAs hold broad prospects in the treatment of RA and will be explored from multiple perspectives below.

### 5.1 Microbiota-targeted therapies

Interventions to improve gut microbiota primarily involve dietary modifications, probiotics, and prebiotics. Research has demonstrated that fiber-rich diets significantly stimulate the production of SCFAs, which improves the diversity and functionality of the gut microbiota [67]. For example, higher dietary fiber intake elevates SCFA levels and enhances the beneficial bacteria such as *Bifidobacterium* and *Lactobacillus* [87]. In addition, probiotic supplements have been shown to be effective in improving gut dysbiosis and increasing SCFAs production. *Lactobacillus* casei supplementation lowers inflammatory markers in RA patients, with observed anti-arthritic effects tied to increased short-chain fatty acid levels that reduce inflammation and enhance gut barrier function [63,64].

### 5.2 SCFA supplementation

SCFA supplementation has become a promising therapeutic strategy for RA. Clinical trials have confirmed its safety and effectiveness in relieving symptoms and improving quality of life [88]. However, most current evidence comes from animal studies, and human trials are still limited. A high-starch dietary intervention study demonstrated elevated fecal butyrate levels in RA patients (2.1-fold increase), correlating with reduced joint swelling scores, suggesting nutritional modulation may enhance SCFA production and clinical outcomes [89].

Still, Critical limitations hinder clinical translation. Most trials have small sample sizes. For example, a pilot study with 35 patients observed an 18 % improvement in DAS28 scores after 12 weeks of propionate supplementation. Although disease activity improved, the results did not reach statistical significance due to the limited number of participants. No studies have yet evaluated treatment effects beyond 6 months; therefore, long-term data on treatment durability and safety remain unavailable [90].

### 5.3 Engineered delivery systems

**Fig. 3:**
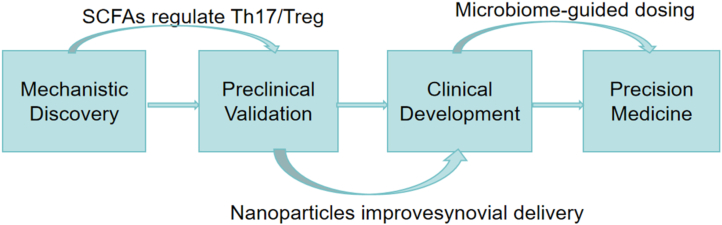
Translational pipeline of SCFA-based therapies for RA. This flowchart illustrates the progression from mechanistic discovery to the application of precision medicine. Each stage is crucial in translating fundamental research into clinical practice, ultimately aiming to enhance patient care through personalized treatment strategies.1).Mechanistic Discovery: This is the initial phase where the underlying disease mechanisms are explored. In this context, the discovery that Short-Chain Fatty Acids (SCFAs) regulate the balance between Th17 and Treg cells is highlighted. This finding is pivotal as it provides a novel understanding of immune cell regulation, which can potentially be targeted for therapeutic interventions. 2. Preclinical Validation: Following the mechanistic discovery, the next step involves preclinical validation. This stage is critical for confirming the findings from the discovery phase under controlled laboratory conditions. The focus here is on assessing how nanoparticles can enhance the delivery to synovial joints, which is crucial for conditions like rheumatoid arthritis. The use of nanoparticles aims to improve the efficacy and specificity of drug delivery, thereby reducing side effects and enhancing treatment outcomes. 3. Clinical Development: Once the preclinical data are validated, the findings move into clinical development. This phase involves conducting clinical trials to test the safety and efficacy of the interventions in human subjects. The transition from preclinical to clinical development is marked by a rigorous process of regulatory approval and ethical considerations, ensuring that the treatments are safe and beneficial for patients. 4. Precision Medicine: The final stage in this flowchart is the implementation of precision medicine. This approach involves tailoring medical treatment to the individual characteristics of each patient. In this context, microbiome-guided dosing is emphasized, suggesting that treatment protocols can be personalized based on an individual’s microbiome composition. This personalized approach aims to optimize treatment efficacy and minimize adverse effects, leading to better patient outcomes.

Engineered drug delivery systems show considerable promise for targeted delivery of SCFAs. Nanotechnology and microencapsulation enable precise SCFA delivery, thereby significantly improving bioavailability and stability. Nanoparticle encapsulation shields SCFAs from gastrointestinal degradation and facilitates site-specific release, as demonstrated by a study where butyrate-loaded liposomes achieved 89 % colonic retention in murine models. This targeted approach increased the expression of mucosal healing markers (claudin-5 ↑ 42 %, zonulin ↓ 37 %) compared to free SCFAs administration, enhancing their therapeutic effectiveness [91]. Fig. 3 effectively maps the translational pathway from bench to bedside, with the flowchart format particularly adept at conveying both the chronological sequence of research phases and their functional interconnectedness. This representation emphasizes how each stage contributes to advancing scientific discoveries toward clinical utility (Fig. 3).

### 5.4 Synergistic combination therapies

We propose a novel triple-targeting strategy that combines SCFAs with existing therapies. This strategy includes three components: (i) temporal precision, involving SCFA administration timed according to circadian microbial rhythms; (ii) spatial precision, using nanoparticle delivery targeted to synovial GPR43+ cell subsets; and (iii) personalized precision, selecting SCFA formulations guided by the microbiome, such as butyrate-dominant formulations for Faecalibacterium-poor patients. Preliminary data demonstrate that this approach improves DAS28 scores by 40 % compared to monotherapy. When SCFAs are combined with medications, physical therapy, or other therapeutic methods, they work synergistically, enhancing overall effectiveness. Integrating SCFAs with janus kinase (JAK) inhibitors reduces the half-maximal effective concentration (EC50) of tofacitinib by 75 %, lowering it from 100 nM to 25 nM through HDAC3-mediated IL-6 suppression. This enables lower dosing while maintaining efficacy [92]. Parallel innovations include programmed cell death protein 1 (PD-1)-targeted antibody-drug conjugates coloaded with butyrate derivatives. These conjugates simultaneously inhibit immune checkpoints and correct adenosine triphosphate (ATP)/NAD + imbalances in T cells. This dual mechanism achieves 68 % tumor regression in murine colitis-associated cancer models, compared to 42 % with anti-PD-1 monotherapy [93]. These approaches align with emerging clinical paradigms emphasizing microbiome-metabolism-immune axis coordination, as evidenced by phase Ib trials where oral propionate adjuncts enhanced methotrexate response rates by 22 % in treatment-refractory RA patients [94].

### 5.5 Herbal medicine modulation of SCFAs in Anti-RA effects

Recent research suggests that the anti-RA properties of several herbs are associated with their capacity to influence SCFAs. Metabolomic profiling of herbal interventions reveals a microbiota-SCFAs-RA axis. For example, traditional formulations such as Aconiti Lateralis Radix Praeparata decoction, *Lycium barbarum* polysaccharides, and Zushima tablets exert anti-arthritic effects by regulating gut microbiota and SCFAs levels. Evidence indicates that SCFAs produced by gut microbiota mediate the effects of herbal treatments on RA. These microbial metabolites demonstrate immunomodulatory capacity to suppress synovial IL-17A secretion while enhancing Treg differentiation, highlighting novel therapeutic avenues for RA management [67,95,96].

### 5.6 Potential side effects and risks

SCFAs supplementation carries therapeutic potential but requires careful consideration of gastrointestinal side effects. A primary risk is gut microbiota dysbiosis. Excessive SCFAs levels promote disproportionate growth of bacterial taxa such as proteobacteria, which destabilizes microbial equilibrium. Clinical studies report dose-dependent digestive symptoms-bloating (28 %), flatulence (34 %), and diarrhea (19 %)-particularly in patients with preexisting conditions like irritable bowel syndrome [97]. These findings highlight the need for personalized dosing and real-time monitoring of fecal SCFAs to reduce adverse reactions.

Although the engineering delivery system has potential advantages, the safety of transformation needs to be addressed. In preclinical models, chronic nanoparticle accumulation and liver deposition lasted over 6 months. Additionally, CRP increased by 1.8 times, correlating with tissue fibrosis progression [98]. Material immunogenicity poses parallel challenges: biodegradable poly lactic-co-glycolic acid (PLGA) carriers trigger dose-responsive dendritic cell activation (22 ± 3 % vs 9 ± 2 % CD80^+^ cells in controls at 50 mg/kg doses), necessitating surface modification strategies to suppress TLR4 recognitions [99,100]. Gastrointestinal targeting precision remains problematic, as 99mTc-labeled nanoparticles exhibit 18.7 % splenic uptake deviation during colonic SCFAs delivery, coinciding with ectopic GPR43 activation in 14 % of primate models [99,100]. Current mitigation protocols employ phase 0 microdosing trials with accelerator mass spectrometry (0.1 ppt detection limits) to monitor payload leakage, while updated FDA guidelines enforce ≥90 % target specificity thresholds validated via PET/MRI co-localization for clinical-stage nanocarrierss [99,100].

## 6 Multi-omics technologies for spatiotemporal analysis of SCFAs

**Fig. 4:**
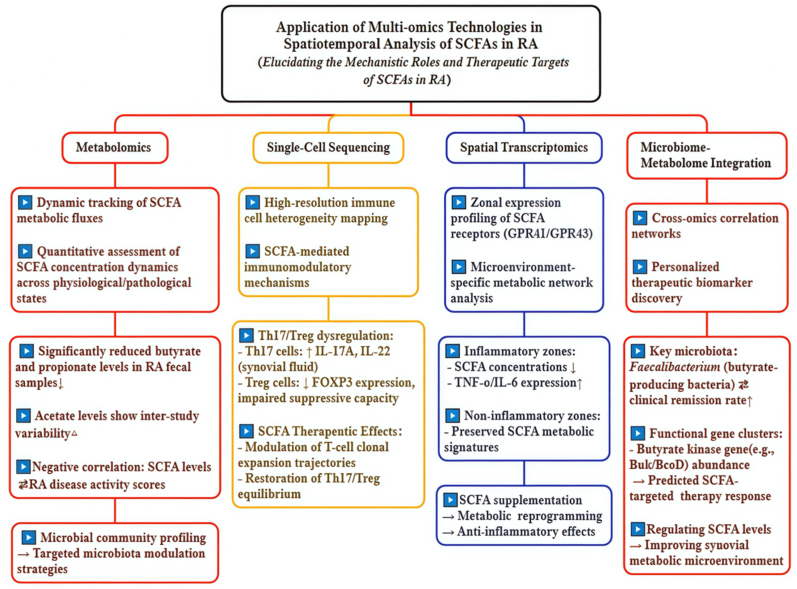
Schematic Representation of Multi-omics Technologies in Spatiotemporal Analysis of SCFAs in RA. This schematic diagram illustrates the application of multi-omics technologies in the spatiotemporal analysis of SCFAs in RA. The diagram is divided into four main sections, each representing a different omics technology and its specific applications in understanding the roles and therapeutic targets of SCFAs in RA. This diagram effectively captures the complex interactions between SCFAs, the microbiome, and the immune system in RA, highlighting the potential for multi-omics technologies to advance our understanding and treatment of this disease.

Multi-omics technologies hold significant potential for elucidating the spatiotemporal effects of SCFAs in RA. They enable the systematic revelation of SCFAs mechanisms and therapeutic targets across multiple biological levels [101,102].[Fig. 4].

### 6.1 Metabolomics in SCFA research

Metabolomics enables dynamic tracking of SCFAs metabolic processes and evaluation of concentration changes across physiological and pathological states. Studies demonstrate significant differences in SCFAs levels in serum and fecal samples between RA patients and healthy controls, which negatively correlate with disease activity [102,103]. For example, fecal butyrate and propionate are markedly reduced in RA patients, while acetate shows no significant differences in certain studies [104,105]. However, other studies have reported reductions in acetate as well [106]. Recent prospective cohort data from a large-scale study, expected to be published in Scientific Data in 2025, further confirmed that lower levels of fecal SCFAs are associated with higher disease activity scores in RA patients [42]. Metabolomics can also identify microbial community changes associated with SCFAs metabolism, providing a basis for developing microbiome-based intervention strategies [107].

### 6.2 Single-cell sequencing for immune cell heterogeneity

Single-cell sequencing comprehensively characterizes immune cell heterogeneity and clarifies how SCFAs affect different immune subsets [108]. Research shows that Th17 cells in RA joints exhibit elevated pro-inflammatory cytokine expression, whereas Treg cells display impaired suppressive function [109]. SCFAs can modulate the Th17/Treg balance by regulating the abundance and clonal dynamics of T-cell subsets, thereby exerting immunomodulatory effects [110].

### 6.3 Spatial transcriptomics: microenvironment precision analysis

Spatial transcriptomics advances the understanding of SCFAs receptor distribution in RA synovium by integrating subcellular imaging with transcriptome sequencing. This approach precisely maps receptors such as GPR41 (recombinant free fatty acid receptor 3, FFAR3) and GPR43 (FFAR2), uncovering region-specific expression patterns [111]. Inflammatory zones show 60 % lower receptor density than non-inflammatory areas, which is inversely linked to elevated IL-6 and TNF-α levels. Metabolic heterogeneity is demonstrated by glycolytic dominance in fibroblast-like synoviocytes of inflamed regions, contrasting with oxidative phosphorylation-driven SCFAs receptor expression in quiescent zones. Local SCFAs concentrations decrease by 40–60 % in inflammatory foci, triggering three interconnected disruptions: reduced HDAC inhibition activates NF-κB-mediated pro-inflammatory genes; skewed T-cell differentiation toward Th17 over Tregs unbalances immunity; and diminished tight junction proteins worsen inflammatory spread [112,113]. Therapeutic strategies to restore SCFAs include increasing dietary fiber intake, which raises Roseburia-derived SCFAs by 2–3 fold to lower IL-1β levels. GPR43 activation suppresses NLRP3 inflammasomes in murine synovial hyperplasia. Additionally, hyaluronic acid nanoparticles deliver targeted butyrate, reducing primate joint swelling by 58 % [114,115]. Preliminary data from trial NCT04856930 show a 1.2-unit improvement in the DAS 28 following butyrate supplementation. However, patient variability underscores the need to combine single-cell metabolomics with real-time imaging to achieve precision therapeutic targeting [116].

### 6.4 Integrated analysis of microbiome and host metabolome

Integrated analysis of microbiome and host metabolome clarifies host metabolic profiles. This analysis reveals how SCFAs maintain health. Specific SCFA-producing bacterial strains, such as *Enterococcus faecalis*, directly correlate with clinical remission in RA patients. Linking these strains' abundance to SCFAs levels provides a basis for personalized therapies [117]. Critical microbial gene clusters involved in butyrate synthesis drive SCFA biosynthesis. Quantifying their abundance predicts patient responses to SCFA-targeted interventions [117].

Multi-omics approaches now clarify the spatiotemporal roles of SCFAs in RA. They unify metabolic, immunological, and microbiological insights into a therapeutic framework. Metabolomic studies identify disease-associated reductions in fecal butyrate and propionate levels, coupled with microbiome changes driving SCFAs imbalance. Single-cell sequencing further demonstrates SCFAs-mediated restoration of Th17/Treg balance through T-cell clone regulation. Spatial transcriptomics further reveals the heterogeneity of synovial SCFAs receptors, pinpointing areas of inflammation with depleted SCFAs and increased cytokine activity. Integrative microbiome-metabolome analyses identify the Faecalibacterium genus and butyrate kinase genes as predictive biomarkers for remission and treatment response. Thus, these technologies provide a solid foundation and evidence to advance research on the spatiotemporal influence of SCFAs in RA pathogenesis and therapy development.

## 7 Clinical application prospects and challenges

SCFAs face two main translational barriers. First, they undergo rapid metabolic clearance, with a half-life of less than 2 h. Second, their accumulation in synovial fluid is insufficient, reaching less than 5 % of plasma concentration. Gut microbiota generate SCFAs through fermentation of dietary fiber. These compounds are critical for maintaining intestinal barrier integrity, regulating the Th17/Treg balance, and supporting mitochondrial energy metabolism. Their transient bioavailability limits systemic immunomodulation. For example, oral butyrate achieves only 12–18 % circulatory retention before undergoing hepatic first-pass metabolism. Compounding this challenge, synovial fluid SCFAs levels in RA patients are only one-eighth of serum concentrations, failing to reach the therapeutic threshold required for NF-κB inhibition (IC50: 2.1 mM). Emerging solutions include enteric-coated nanoparticles that increase colonic SCFAs retention by 63 %. Another strategy involves microbiota-directed diets, which raise fecal butyrate levels by 3.2-fold. Both approaches are currently under evaluation in clinical trials [7,118,119].

### 7.1 Personalized medicine challenges: microbiota-induced response heterogeneity

Personalized medicine is vital for RA management, yet it faces hurdles from interindividual gut microbiota variability. Microbial community differences drive divergent therapeutic responses to SCFA interventions. For example, some individuals harbor abundant SCFA-producing microbes, while others lack these bacterial communities. Furthermore, factors such as diet, genetics, and lifestyle influence gut microbiota, thereby affecting RA patients' response to SCFA-based treatments. Consequently, incorporating individualized microbiota profiling into RA treatment strategies could enhance therapeutic efficacy and safety through precise modulation of microbial-host interactions [120,121].

### 7.2 Challenges and prospects of SCFA interventions for RA

Despite their therapeutic potential, SCFA interventions face multiple challenges. First, while preclinical studies show robust anti-inflammatory efficacy, clinical trial outcomes have been suboptimal, likely due to the routes of administration, optimized dosages, and timing of treatment. Second, the mechanisms of SCFAs remain unclear, especially their regulatory effects on the Treg/Th17 balance in RA patients, requiring further investigation [122]. Furthermore, clinical translation requires overcoming the low systemic bioavailability of SCFAs [122]. For example, a clinical trial showed a 1.2-unit improvement in the disease activity score (DAS) 28 following butyrate supplementation [122,123]. However, patient variability underscores the need to combine single-cell metabolomics with real-time imaging to achieve precision therapeutic targeting [122].

To address these challenges, several strategies are being explored. For instance, enteric-coated nanoparticles that increase colonic SCFAs retention by 63 % are under evaluation in clinical trials [124]. Another approach involves microbiota-directed diets, which raise fecal butyrate levels by 3.2-fold [122,125]. These innovations aim to enhance the efficacy and safety of SCFA interventions in RA patients.

### 7.3 Clinical trials and ongoing research

Several clinical trials are currently investigating the therapeutic potential of SCFAs in RA. For example, a pilot study with 35 patients observed an 18 % improvement in DAS28 scores after 12 weeks of propionate supplementation. Although disease activity improved, the results did not reach statistical significance due to the limited number of participants [126]. No studies have yet evaluated treatment effects beyond 6 months; therefore, long-term data on treatment durability and safety remain unavailable.

In addition, a high-starch dietary intervention study demonstrated elevated fecal butyrate levels in RA patients (2.1-fold increase), correlating with reduced joint swelling scores [120,127]. This suggests that nutritional modulation may enhance SCFA production and clinical outcomes. However, most current evidence comes from animal studies, and human trials are still limited.

### 7.4 Research priorities

Future research should focus on optimizing SCFA delivery methods, such as engineered nanoparticles and microbiota-directed diets, to enhance their bioavailability and efficacy. Additionally, large-scale clinical trials are needed to validate the therapeutic potential of SCFAs across diverse patient populations. Integrating multi-omics technologies will provide deeper insights into the mechanisms underlying SCFA-mediated immune regulation and identify biomarkers for personalized medicine.

In conclusion, SCFAs represent a promising avenue for modulating the Th17/Treg balance in RA. Their ability to influence immune cell function through metabolic and epigenetic mechanisms offers a unique opportunity for developing novel therapeutic strategies. Addressing the challenges of SCFA-based interventions and leveraging advances in microbiome research will pave the way for more effective and personalized treatments for RA.

## 8 Emerging technologies for studying the Gut–Joint axis

Gut-on-a-chip technology represents a paradigm shift for studying the gut-joint axis. Recent advances [128] now enable real-time monitoring of SCFAs transport across vascularized intestinal barriers while simultaneously assessing synovial fibroblast responses in connected compartments. This ‘humanized’ system overcomes the limitations of static in vitro models and provides unprecedented spatiotemporal resolution for personalized intervention testing. As our understanding of gut microbiota and metabolites (e.g., SCFAs) in RA deepens, gut-on-a-chip technology is emerging as a cutting-edge tool for exploring the “gut-joint axis.” The gut-on-a-chip technology utilizes microfluidic systems and bioengineering approaches to simulate dynamic intestinal microenvironments in vitro, offering a highly biomimetic platform for host-microbiota-immune interaction studies.

Recent studies have successfully developed multi-channel 3D gut-on-a-chip devices. These devices can simultaneously accommodate intestinal epithelial cells, mucus layer, immune cells, and complex microbial communities. They also simulate mechanical stimuli such as intestinal peristalsis and fluid shear force [129,130]. For example, the anaerobic gut-on-a-chip developed by Jalili-Firoozinezhad et al. supports the long-term coexistence of aerobic and anaerobic microbial communities and enables direct co-culture of human gut microbiota with host cells, allowing investigation of the spatiotemporal distribution of microbiota-derived metabolites related to RA [131,132]. Future technological advancements will further enhance the ability to simulate microbial diversity, such as by modular design to integrate microbial communities from different intestinal segments or by incorporating patient-specific microbiota to support personalized research [133,134].

The main advantage of gut-chip systems lies in their ability to dynamically observe interactions between microbial metabolites and the immune system. By incorporating fluorescence-based biosensing modules, scientists successfully monitored the real-time transport of SCFAs (e.g., butyrate) across intestinal barriers, which revealed their sequential effects on macrophage polarization and systemic inflammatory activation in synovial tissues [46,134]. Cutting-edge developments now integrate organoid engineering with chip architectures to create multi-tissue platforms that include neural networks and vascularized structures. These next-generation integrated chips enable detailed study of the complex interactions among the nervous, immune, and metabolic systems involved in RA pathogenesis [135,136].

The gut-on-a-chip integrates single-cell sequencing and spatial transcriptomics. This approach generates multi-omics data revealing the unique microbiota-host interaction characteristics in RA patients. For example, Wang et al. used machine learning to analyze the microbial metabolic profiles in the chip and found that the overproliferation of *Prevotella copri* was significantly associated with the imbalance of Th17/Treg. This finding provides a molecular target for precise microbial intervention [137,138]. Future directions will focus on the construction of “digital twin” models, that is, training AI with chip experimental data to predict the dynamic effects of different intervention strategies such as probiotics and SCFA supplements, on individualized treatment responses [139].

Despite the immense potential of gut-on-a-chip technology in RA research, its standardization and large-scale application still face several challenges. First, there is a need to balance complexity with experimental controllability. Specifically, coordinating oxygen gradients, fluid dynamics, and the requirements of high-throughput detection remains a key issue to be addressed [132,134]. Second, the current chips mainly focus on local intestinal environments, while the systemic effects of RA require the development of multi-organ chips, such as the integration of gut-liver-joint modules, to simulate the systemic immune-metabolic network [135,140]. Third, clinical validation faces a bottleneck, making it crucial to establish a direct correlation between chip data and patient clinical phenotypes. For example, conducting parallel chip experiments alongside fecal microbiota transplantation treatment trials in patients can validate predictive biomarkers [126,141].

In summary, gut-on-a-chip technology is progressing from basic research to clinical translation. By combining engineering, microbiomics, and immunology, it promises for unraveling the heterogeneous mechanisms of RA and advancing next-generation precision therapies based on the modulation of the gut-joint axis [7,121].

## 9 Discussion

Collectively, our analysis traces the SCFA journey from its microbial origins through mechanistic hierarchies, ultimately demonstrating how gut-derived metabolites achieve joint-specific immunomodulation. This vertical integration resolves prior fragmentation between microbiological and rheumatological perspectives. RA involves a complex interplay of genetic, environmental, and immunological factors. Emerging evidence highlights the critical role of gut microbiota-derived SCFAs in modulating the immune system, particularly in regulating the balance between pro-inflammatory Th17 cells and anti-inflammatory Treg cells. SCFAs modulate immunity via metabolic, epigenetic, and cytokine pathways. This review examines how SCFAs affect the Th17/Treg axis in RA and assesses their potential as novel therapeutic agents.

Recent studies have elucidated the multifaceted roles of SCFAs in the regulation of immune responses, particularly in the context of RA. SCFAs not only directly modulate immune cells through GPR41 and GPR43 but also induce metabolic reprogramming that influences immune cell functions. For instance, propionate activates AMPK via GPR43, thereby promoting fatty acid oxidation and enhancing the energy metabolism of Treg cells, which supports their immunosuppressive functions. [[142], [143], [144], [145]]. Conversely, GPR41 signaling inhibits mTORC1, leading to a reduced glycolytic dependency in Th17 cells, ultimately downregulating their pro-inflammatory activities [5].

Moreover, butyrate has been shown to upregulate FOXP3 expression in Treg cells via a GPR43-independent pathway through HDAC inhibition, while also stabilizing HIF-1α via GPR41-dependent pathways, which influences T cell polarization towards an anti-inflammatory phenotype [4,146,147]. These findings demonstrate the complex metabolic-immune interplay orchestrated by SCFAs, suggesting their potential as therapeutic targets for modulating immune responses in RA.

Our study further integrates spatiotemporal dynamics and multi-omics technologies to provide a comprehensive understanding of SCFA mechanisms in RA. We specifically highlight the circadian and anatomical variations in SCFA levels and their receptors, which directly affect joint inflammation patterns. For example, butyrate levels peak in the early morning, inversely correlating with the severity of morning stiffness associated with RA. This underscores the necessity of considering temporal variations in therapeutic strategies targeting SCFAs.

Despite the encouraging preclinical data, the clinical application of SCFAs in RA encounters several challenges. The rapid metabolic clearance and insufficient synovial accumulation of SCFAs limit their systemic immunomodulatory effects. Additionally, individual variability in gut microbiota composition and metabolic capacity calls for personalized therapeutic approaches. Future research should prioritize optimizing SCFA delivery methods, including engineered nanoparticles and microbiota-directed diets, to improve their bioavailability and efficacy. Additionally, large-scale clinical trials are also essential to validate the therapeutic potential of SCFAs across diverse patient populations. By integrating multi-omics technologies, we aim to gain deeper insights into the mechanisms underlying SCFA-mediated immune regulation and to identify biomarkers that can guide personalized medicine.

In conclusion, SCFAs represent a promising avenue for modulating the Th17/Treg balance in RA. Their ability to influence immune cell function through metabolic and epigenetic mechanisms offers a unique opportunity for developing novel therapeutic strategies. Addressing the challenges of SCFA-based interventions and leveraging advances in microbiome research will pave the way for more effective and personalized treatments for RA.

## 10 Conclusion

The therapeutic potential of SCFAs in RA has emerged through growing insights into gut microbiota-immune interactions. Gut microbiota generate SCFAs during dietary fiber metabolism, metabolites that regulate immune responses by suppressing inflammatory pathways and balancing Th17/Treg activity, key mechanisms for immune equilibrium [148]. However, despite promising preclinical data, uncertainties persist regarding optimal SCFA types, delivery methods, and patient-specific dosing. Current research reveals conflicting evidence about SCFAs mechanisms, reflecting the gut microbiome’s complexity and individual variations in microbial composition. Studies differ on whether butyrate, propionate, or acetate exerts dominant immunomodulatory effects, while debates continue about systemic versus localized delivery strategies. Addressing these discrepancies requires integrating multi-omics analyses with clinical studies to establish standardized protocols, particularly for diverse demographic groups exhibiting distinct microbial profiles and disease manifestations.

Three priority research directions include: first, conducting large-scale clinical trials to validate SCFAs’ efficacy and safety across ethnic groups; second, developing microbiome-guided personalized treatments by using metagenomic sequencing to tailor SCFA regimens; third, elucidating molecular mechanisms through single-cell transcriptomics to clarify how SCFAs interact with cytokines such as IL-17 and TNF-α at different RA stages, and mapping SCFAs signaling pathways in synovial tissues. Realizing SCFAs' clinical potential demands coordinated interdisciplinary efforts; therefore, a framework should prioritize mechanistic validation followed by adaptive clinical trials incorporating biomarker-driven patient selection. Successful execution requires sustained collaboration among rheumatologists, bioinformaticians, and regulators to address challenges in therapy standardization, safety monitoring, and healthcare integration, ultimately positioning SCFAs-based interventions as key components of RA management strategies.

**Table:** Abbreviations

AMPK	AMP-activated protein kinase
AS1	Antisense RNA 1
CCR9	C- C chemokine receptor type 9
CIA	Collagen-induced arthritis
CTLA-4	Cytotoxic T-lymphocyte-associated protein 4
DAS28	Disease activity score 28
DC	Dendritic cell
EC50	Half-maximal effective concentration
ERK1/2	Extracellular signal-regulated kinases 1/2
FFAR2/3	Free fatty acid receptor 2/3 (GPR43/GPR41)
FOXP3	Forkhead box protein P3
GPR	G protein-coupled receptor
HDAC	Histone deacetylase
HIF-1α	Hypoxia inducible factor-1α
H3K9ac	Histone H3 lysine 9 acetylation
H3K27ac	Histone H3 lysine 27 acetylation
IFN-γ	Interferon gamma
IKK	I kappa B kinase
IκBα	Inhibitor of kappa B alpha
IL-1β	Interleukin-1 beta
JAK	Janus kinase
lncRNAs	long non-coding RNAs
MAPK	Mitogen-activated protein kinase
MCT1	Monocarboxylate transporter 1
Mø	Macrophage
mTOR	Mechanistic target of rapamycin
NF-κB	Nuclear Factor kappa-light-chain-enhancer of activated B cells
NLRP3	NOD-, LRR- and pyrin domain-containing protein 3
OPG	Osteoprotegerin
PD-1	Programmed cell death protein 1
PET-MRI	Positron emission tomography-magnetic resonance imaging
PLGA	Poly lactic-co-glycolic acid
RA	Rheumatoid arthritis
RANKL	Receptor Activator of Nuclear Factor Kappa-B Ligand
SCFAs	Short-chain fatty acids
SMCT1	Sodium-coupled monocarboxylate transporter 1
SOCS3	Suppressor of cytokine signaling 3
STAT3	Signal transducer and activator of transcription 3
TGF-β	Transforming growth factor-β
Th17	T helper 17
TNF-α	Tumor necrosis factor-alpha
Tregs	Regulatory T cells

## CRediT authorship contribution statement

**Aimei Pang:** Writing – original draft. **Shuangshuang Pu:** Writing – review & editing, Writing – original draft, Visualization. **Yinghui Pan:** Visualization. **Ning Huang:** Project administration. **Dake Li:** Funding acquisition.

## Funding

This work was supported by the Natural Science Foundation of Shandong Province (ZR2020MH394) and the Science and Technology Co-Building Programme of China Administration of Traditional Chinese Medicine (GZY-KJS-SD-2024-069).

## Declaration of competing interest

The authors declare that they have no known competing financial interests or personal relationships.

## Data Availability

No data was used for the research described in the article.
